# Reliability and Validity of a New Taekwondo-Specific Change-of-Direction Speed Test With Striking Techniques in Elite Taekwondo Athletes: A Pilot Study

**DOI:** 10.3389/fphys.2022.774546

**Published:** 2022-04-26

**Authors:** Ali Aloui, Amel Tayech, Mohamed Arbi Mejri, Issam Makhlouf, Cain C. T. Clark, Urs Granacher, Hassane Zouhal, Abderraouf Ben Abderrahman

**Affiliations:** ^1^ M2S (Laboratoire Mouvement, Sport, Santé)—EA 1274, University of Rennes, Rennes, France; ^2^ High Institute of Sport and Physical Education of Ksar-Saïd, Manouba University, Tunis, Tunisia; ^3^ Tunisian Research Laboratory “Sports Performance Optimization”, National Center of Medicine and Science in Sports (CNMSS), Tunis, Tunisia; ^4^ Centre for Intelligent Healthcare, Coventry University, Coventry, United Kingdom; ^5^ Division of Training and Movement Sciences, University of Potsdam, Potsdam, Germany; ^6^ Institut International des Sciences du Sport (2I2S), Irodouer, France

**Keywords:** taekwondo-specific testing, sport-specific performance, striking combat sports, sensitivity, taekwondo electronic scoring system

## Abstract

The purpose of this study was to examine the test-retest reliability, and convergent and discriminative validity of a new taekwondo-specific change-of-direction (COD) speed test with striking techniques (TST) in elite taekwondo athletes. Twenty (10 males and 10 females) elite (athletes who compete at national level) and top-elite (athletes who compete at national and international level) taekwondo athletes with an average training background of 8.9 ± 1.3 years of systematic taekwondo training participated in this study. During the two-week test-retest period, various generic performance tests measuring COD speed, balance, speed, and jump performance were carried out during the first week and as a retest during the second week. Three TST trials were conducted with each athlete and the best trial was used for further analyses. The relevant performance measure derived from the TST was the time with striking penalty (TST-TSP). TST-TSP performances amounted to 10.57 ± 1.08 s for males and 11.74 ± 1.34 s for females. The reliability analysis of the TST performance was conducted after logarithmic transformation, in order to address the problem of heteroscedasticity. In both groups, the TST demonstrated a high relative test-retest reliability (intraclass correlation coefficients and 90% compatibility limits were 0.80 and 0.47 to 0.93, respectively). For absolute reliability, the TST’s typical error of measurement (TEM), 90% compatibility limits, and magnitudes were 4.6%, 3.4 to 7.7, for males, and 5.4%, 3.9 to 9.0, for females. The homogeneous sample of taekwondo athletes meant that the TST’s TEM exceeded the usual smallest important change (SIC) with 0.2 effect size in the two groups. The new test showed mostly very large correlations with linear sprint speed (*r* = 0.71 to 0.85) and dynamic balance (*r* = −0.71 and −0.74), large correlations with COD speed (*r* = 0.57 to 0.60) and vertical jump performance (*r* = −0.50 to −0.65), and moderate correlations with horizontal jump performance (*r* = −0.34 to −0.45) and static balance (*r* = −0.39 to −0.44). Top-elite athletes showed better TST performances than elite counterparts. Receiver operating characteristic analysis indicated that the TST effectively discriminated between top-elite and elite taekwondo athletes. In conclusion, the TST is a valid, and sensitive test to evaluate the COD speed with taekwondo specific skills, and reliable when considering ICC and TEM. Although the usefulness of the TST is questioned to detect small performance changes in the present population, the TST can detect moderate changes in taekwondo-specific COD speed.

## 1 Introduction

Taekwondo is a combat sport classified as an activity of an intermittent nature, of high physiological intensity, with motor actions executed at high speed, mainly of the lower limbs (Bridge et al., 2014; Gaamouri et al., 2019; da Silva Santos et al., 2020; Ojeda-Aravena et al., 2021). The physical and physiological demands and specificity of taekwondo competition require athletes to be efficient in different aspects of physical fitness, including aerobic and anaerobic power, muscular strength and power, flexibility, speed, and agility. (Marković et al., 2005; Bouhlel et al., 2006; Bridge et al., 2014). This sport has undergone many regulatory changes over the preceding decades, and, as a consequence of this dynamism, the inclusion of new electronic scoring systems (electronic body protector and headgear) is now ubiquitous. During taekwondo matches, over an area of 16 m^2^, fighters use quick displacement chained by various types of dodges with change-of-direction (COD), powerful movements for attacking and counterattacking the opponent’s his/her torso and head, including punching, complex unipedal standing and jumping kicks, and defensive actions with hands and feet (cuts, blocks) (Singh et al., 2017; Menescardi et al., 2019; da Silva Santos et al., 2020; Janowski et al., 2020).

The ability to quickly change directions, also known as COD speed, is a fundamental physical attribute in striking combat sports (Brughelli et al., 2008; Young et al., 2015; Chaabene et al., 2018a, 2020). In taekwondo, throughout the combat, fighters need the ability to accelerate, decelerate, and quickly change direction for attacking or reposting, and place themselves in a good position with respect to the opponent to execute various strikes (punches and kicks) with the required speed and accuracy using both sides of their body (Singh et al., 2017; Ojeda-Aravena et al., 2020). Therefore, COD speed is an essential motor ability for successful performance in taekwondo (Marković et al., 2005).

Sports performance studies have more often assessed COD ability through measurements of total time to complete a variety of COD tests (Nimphius et al., 2018). Each COD speed test varies in length, number and angle of direction changes, and travel patterns (Brughelli et al., 2008; Nimphius et al., 2018). Recently, many sport-specific COD speed tests with specific tasks have been developed to evaluate the ability to quickly COD in conjunction with specific motor actions and skills (Chaabene et al., 2018a; Chtara et al., 2020; Makhlouf et al., 2022). Relatively few studies have examined the COD speed capability of taekwondo athletes using field-based general testing methods, including side step and 50-m (10-m × 5-m) shuttle run sprint tests, T-test, modified T-Test, etc. (Bridge et al., 2014). The field-based tests used to assess COD speed in the literature may be challenged on the grounds that they lack mechanical specificity to many of the technical and tactical actions performed in the sport. To this end, only one study has attempted to incorporate COD speed (planned agility) assessment that is more specific to the technical actions performed in taekwondo (Chaabene et al., 2018a). Recently, Chaabene et al. (2018a) examined the specific COD speed capability of taekwondo athletes using a new planned agility test, known as “taekwondo-specific agility test”. The authors reported that the taekwondo-specific agility test is a valid test with high relative and absolute reliability [intraclass correlation coefficient (ICC) = 0.97, typical error of measurement expressed as a coefficient of variation (TEM %) < 5%], as well as a very good ability to detect small and meaningful performance changes [TEM < smallest important change (SIC)]. Moreover, this tool was able to discriminate taekwondo athletes of different competitive level (top-elite vs. elite). Despite this, the Taekwondo-specific agility test has some limitations, such as the examination of only one parameter (i.e., time performance). There may also be limitations in evaluating the skills associated with the combat, which include focusing only on one skill (i.e., roundhouse kick). Indeed, before the changes in the rules, the roundhouse kick “Bandal-Chagi” was the most frequently applied technique; however, after the rule’s modification, its usage decreased by 43% while the quantity of different striking techniques used increased (da Silva Santos et al., 2020). It is generally accepted that taekwondo became, and is, famous for the great variety in striking techniques (Kwok and Cheung, 2021). Although the use of wearable protection devices for the head, body, hands and feet, which reliably measure the power of striking by means of sensors and electronic chips, has changed the paradigm of taekwondo from a game to an objective, qualitative, and scientific sport (Sevinç, 2017; Sevinç and Çolak, 2019; Cho et al., 2020; Janowski et al., 2020; Park et al., 2021), Chaabene et al. (2018a) did not use this electronic scoring system to reliably count kicks scored during Taekwondo-specific agility test performance. During the Taekwondo-specific agility test, the roundhouse kicks were only projected on kick-targets held by partners, at the torso height of the tested athlete.

It has been reported that the electronic body protector and scoring system (PSS) not only protects taekwondo players against injury, but also results in more reliable and accurate scoring (Del Vecchio et al., 2011; Park et al., 2021). On the other hand, it has been well-documented that high-performance taekwondo athletes have high strength and/or power output in short dynamic multi-joint actions during competition, such as displacements in any directions, kicks and punches (da Silva Santos et al., 2020). Moreover, during international taekwondo competitions (matches organized in three rounds of 2 minutes each), ∼80% of the attacks were reportedly applied to the trunk and ∼19% were applied to the head (da Silva Santos et al., 2020; Kwok and Cheung, 2021). Kwok and Cheung (2021) noted that elite athletes executed the highest number of attacks in each match, compared to the sub-elite athletes (41.5 ± 9.7 vs. 34.4 ± 8.2 attacks (mean ± SD), respectively). The results of the above study suggest that ∼52% of attacks are performed by the front leg and ∼47% by the rear leg.

As shown in various sport disciplines, rule changes affect exercise response to competition tasks, and, consequently, the discipline-specific tests (Janowski et al., 2020). Thereby, the choice of an appropriate field-based test for a sport or other physical activity must be centered on the specificity principle and the requirements of the sport or activity to be assessed (Pauole et al., 2000; Chaabene et al., 2018b). Therefore, the purpose of this study was to: 1) examine the reliability and validity of a new taekwondo-specific COD speed test with striking techniques (TST); 2) establish its relationship with linear sprint, muscle power and balance capabilities; and 3) assess whether this taekwondo-specific COD speed test is sensitive and can discriminate between taekwondo athletes of different competitive levels (i.e., top-elite and elite). With reference to the relevant literature (Arabaci et al., 2010; Chaabene et al., 2018a, 2020), we hypothesize that the TST will yield high test-retest reliability, sensitivity, and validity, as well as a meaningful association with sports performance indicators.

## 2 Materials and Methods

### 2.1 Experimental Approach to the Problem

The relative and absolute reliability and the validity of the TST were evaluated using elite taekwondo athletes. The reliability of the TST was verified by means of test-retest trials separated by 7 days. As established by Chaabene et al. (2018a, 2018b), the ecological validity of the TST was based on the specialized scientific literature and considered the displacement and the most-frequently executed striking techniques performed during taekwondo competitions (Moenig, 2017; Sevinç, 2017; Sevinç and Çolak, 2019; Cho et al., 2020; World Taekwondo, 2020), as well as the new PSS (Moenig, 2017; Sevinç, 2017; Cho et al., 2020; World Taekwondo, 2020) planned to be used during the postponed 2021 Olympics. Convergent validity was assessed by comparing TST performance with performance in a COD speed test (i.e., modified COD T-test). The modified COD T-test is a widely applied test in taekwondo (Marković et al., 2005; Monks et al., 2017; Chaabene et al., 2018a, 2020; Taskin and Akkoyunlu, 2020). In addition, the modified COD T-test has frequently been used in studies which aim to assess the validity of a novel COD speed test in many sports such as handball, soccer, basketball, taekwondo, fencing. It has previously been reported that the modified COD T-test is feasible, valid, and reliable (Sassi et al., 2009; Chaabene et al., 2018a; Chtara et al., 2020). To test the relationship of the TST performances with proxies of athletic performance of taekwondo athletes, linear sprint (5, 10, 20, and 30-m), muscular power (squat and countermovement jumps, single-leg hop, single-leg triple hop, and 5-jump), and balance (standing stork balance and Y-balance) tests were selected. The discriminatory ability of the TST was examined based on results from national and international (top-elite) and national (elite) taekwondo championships.

### 2.2 Participants

Chaabene et al. (2018a) reported a correlation of 0.71 between a taekwondo-specific agility test and the T-Test. Thus, in this study, to be conservative, a very large correlation coefficient (*r* = 0.71) was used for the a priori sample size calculation. In order for this study to meet the ethical standards of the journal and based on data from Chaabene et al. (2018a), an a priori power analysis, using G*Power version 3.1.9.7, revealed a required sample size of 18 subjects for our study (correlation: bivariate normal model, pH1 = 0.71, alpha = 1%, power = 80%). Therefore, to meet this requirement, accounting for potential loss to attrition, twenty elite taekwondo athletes including 10 males (age, 17.9 ± 2.4 years; height, 178.3 ± 5.1 cm; body mass, 64.4 ± 5.8 kg; %body fat: 13.7 ± 2.2%; training background, 9.5 ± 1.2 years; mean ± SD) and 10 females (age, 15.6 ± 0.8 years; height, 167.5 ± 5.9 cm; body mass, 55.0 ± 8.3 kg; %body fat: 23.5 ± 1.8%; training background, 8.3 ± 1.3 years; mean ± SD) belonging to the Tunisian taekwondo national team voluntarily participated in this study. They were regularly competing at a national, for >6 years, and international level, for >3 years. The weekly training program during the competitive period included nine training sessions in 7 days, and a free day on Sunday. Each training session lasts about 2 hours. All participants were free of injuries and neuromuscular problems in the last 10 weeks prior to the start of the study, and were not in a period of body mass reduction (Ojeda-Aravena et al., 2021). Since championships participation is a criterion for the classification of athletes (Chaabene et al., 2018a; Tayech et al., 2020, 2022; Makhlouf et al., 2022), based on their international and national taekwondo championships participation, 10 athletes (compete at national and international level) were included in the top-elite (five males and five females: age, 16.4 ± 0.8 years; height, 173.8 ± 7.9 cm; body mass, 59.6 ± 9.7 kg; %body fat: 18.5 ± 5.9%; training background, 8.4 ± 1.3 years; mean ± SD) and 10 athletes (compete at national level) in the elite (five males and five females: age, 18.1 ± 2.3 years; height, 172 ± 7.8 cm; body mass, 60.2 ± 7.5 kg; %body fat: 18.1 ± 2.3%; training background, 9.4 ± 1.3 years; mean ± SD) subgroups. After receiving a thorough explanation of the protocol, athletes/legal representatives gave written consent to participate in this study. The study was conducted in accordance with the declaration of Helsinki (World Medical Association, 2013) and the protocol was fully approved by the ethics committee of the National Center of Medicine and Sciences in Sport of Tunis, Tunisia before the commencement of the assessments.

### 2.3 Experimental Design

At the competitive phase of the taekwondo season, and during 2-week preceding the experiment, participants were familiarized (two sessions/week) with the general environment, equipment, form and technique of each fitness test to minimize learning effects during the course of the experiment. During this time, assessors demonstrated the proper form and mechanics of movement for the execution of all tests and explained the key technical features. Anthropometrical measurements were also determined for each participant 2 days before the start of the experiment, in the fasting state. Body mass was measured to the nearest 0.1 kg using an electronic scale (LifeSource Model UC-321P; A&D Company, Tokyo, Japan). Height was measured to the nearest 0.1 cm using a wall-mounted stadiometer (Easy Glide Stadiometer; Perspective Enterprises, Portage, MI, United States). Participants performed tests during first week and retests during second week in order to examine its reliabilities. Participants’ performance was assessed using log transformed data of reliability statistics of generic performance tests assessing COD speed (i.e., modified COD T-test), balance, speed, and jump performance, for males and females, separately. Data are presented in the Supplementary Material. The same test battery was applied in test and retest. During each week, the tests were conducted on 3 days with a recovery period of 48 h in between. Testing was always conducted indoors, on the taekwondo mat, at the same time-of-day (between 4 p.m. and 6 p.m.) and with the same test sequence, by the same assessors and with similar environmental conditions (temperature ∼18°C and ∼48% humidity). On the first day, participants completed the standing stork balance test, squat jump, countermovement jump, single-leg hop tests, and modified COD T-test. On the second day, each athlete completed the Y-balance test, single-leg triple hop test, and 5-jump test. The remaining tests [linear sprint tests (5, 10, 20, and 30-m), and TST] were conducted on the third day. The rest intervals between each test and within each test session were at least 5 min to allow adequate recovery. Prior to all tests, a 15-min standardized general warm-up (jogging, squatting, jumping, and static and ballistic stretching) was conducted (Tayech et al., 2019, 2020). Prior to the TST, the standardized general warm-up was completed with a specific warm-up (Tayech et al., 2019). Participants performed a set of two sub-maximal repetitions of each test to get prepared for the test. During all tests, instructor-to-participant ratio was 1:1 (Makhlouf et al., 2022). Participants were previously instructed to give their maximum effort during the assessments. Standard verbal encouragement was consistently given for all participants throughout the tests by the same assessors. Before the experiment, all participants were instructed to sleep for 7–8 h before each assessment session (Mejri et al., 2015, 2016) and not to modify their usual diet and hydration habits during the days prior to the assessments, as well as to avoid eating at least 3 hours before each testing session (Ojeda-Aravena et al., 2021).

### 2.4 Testing Procedures

#### 2.4.1 Taekwondo Specific Change-of-Direction Speed Test With Striking Techniques

**FIGURE 1:**
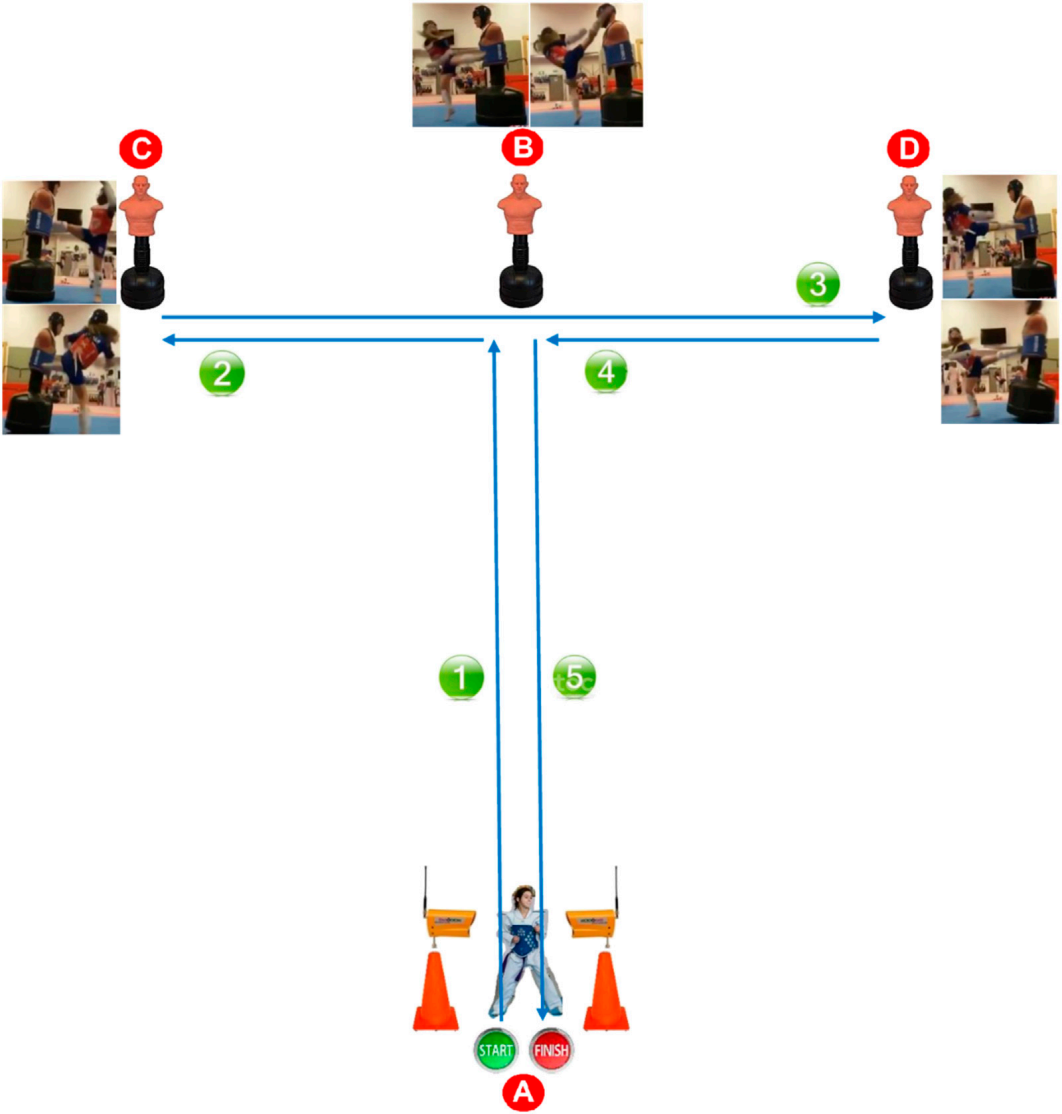
Taekwondo specific change-of-direction speed test with striking techniques (TST).

The TST course is similar to the modified COD T-test, except that front and back sprints during the modified COD T-test were replaced by front and back lateral shifts during the TST (Figure 1). From a regulatory fight guard position (i.e., feet apart at an anteroposterior distance equivalent to the width of the shoulders and with the right leg positioned behind), with both feet behind the start/finish line (A), the participant is expected to: 1) move forward (left leg forward) towards the dummy (B), in guard position, with chasing steps and without crossing feet as fast as possible, and finish with a right direct-punch projected on the center of an electronic body protector (TK-Strike protector, daedo, Barcelona, Spain) carried by a dummy; 2) turn and move towards the dummy (C) with a lateral shift with chasing steps (left leg forward) and perform a left front-leg side kick (i.e., in taekwondo terminology referred to “Yop-Chagi”) projected on the center of the dummy trunk, followed by a second roundhouse kick with the right leg (in taekwondo terminology referred to “Bandal-Chagi”) projected on the right side of the dummy trunk; 3) change direction, turn and move towards the dummy (D) by adopting also a lateral shift with chasing steps (right leg forward) and perform also a right front-leg side kick (i.e., in taekwondo terminology referred to “Yop-Chagi”) projected on the center of the dummy trunk, followed by a second roundhouse kick with the left leg (in taekwondo terminology referred to “Bandal-Chagi”) projected on the left side of the dummy trunk; 4) change direction, turn and return to the center, moving towards the dummy (B) by adopting also a lateral shift with chasing steps (left leg forward) and perform roundhouse kick (in taekwondo terminology referred to “Bandal-Chagi”) with a leg of the participant’s choice (right or left) projected on an electronic headgear (Gen-2 E-Headgear, TK-Strike-Protector, Daedo, Barcelone, Espagne) worn by the dummy, at the same level of the participant’s head followed by a second back kick with the other leg (in taekwondo terminology referred to “Dwit-Chagi” (Guimarães et al., 2020) projected on the center of the dummy trunk; 5) move back to the start/finish line (A) in a guard position, by adopting also a lateral shift with chasing steps and without crossing feet.

If the participant crosses his or her feet during the lateral shift with chasing steps or falls to the ground during the execution of the different kicks, the trial was instantly stopped and restarted after a 3-min recovery period. The height of the dummy was adjusted at the same height of the participant. The use of electronic scoring equipment (electronic body protector and headgear) during the TST was intended to rationally and objectively count the valid strikes (Moenig, 2017; Sevinç and Çolak, 2019; Tayech et al., 2019, 2020; Cho et al., 2020; Janowski et al., 2020; World Taekwondo, 2020). This generation-2 of the PSS (PSS-G2) only detects strikes to valid scoring areas of the body protector and headgear, and eliminates the “questionable” techniques, providing more valid scores (Moenig, 2017; Sevinç and Çolak, 2019; Cho et al., 2020; Janowski et al., 2020; World Taekwondo, 2020). The sensors in the body protector and headgear and all-around of the foot sensor socks worn by the participants automatically transmit the score (i.e., the number of validated kicks) to the computer screen when they receive sufficiently strong pressure together with correct technique (i.e., according to the sex, category and body mass of the athletes). Participants are familiar with the PSS-G2, because this system is commonly used during training, as well as at local, state, national, and international taekwondo championships. In research and applied work, the use of total time as an indicator of COD performance has been widely considered a valid measure of performance (Nimphius et al., 2018). Therefore, according to the specific performance quantification method used by Farhani et al. (2019), the TST performances are: 1) the time needed to complete the test (TST-TO) (s) [assessed with an electronic timing system (Brower Timing Systems, Salt Lake City, UT, United States)], and 2) the time needed with striking penalty (TST-TSP) (s) (the sum of the TST-TO and the number of non-scored strikes multiplied by 0.5 s). This time-penalty for missed kicks was utilized, given that the mean time of a kick execution is ∼0.5 s among experienced taekwondo athletes (Guimarães et al., 2020). The total number of strikes that should be scored during the TST is seven strikes. Participants were asked to wear their official protectors (i.e., headgear, body protector, groin guard, gloves, teeth protector, shin and forearm guards, and sensing socks) during the test, as in official taekwondo competition (Tayech et al., 2019, 2020). Three trials were recorded for each athlete with the best trial included in the statistical analysis. The TECHN-INDEX refers to the time required to complete striking techniques. It was calculated by deducing the modified COD T-test score from the TST-TO score (Fiorilli et al., 2017; Conte et al., 2020).

### 2.5 Modified Change of Direction T-Test

The modified COD T-test has been described as a measure of planned four-directional agility and body control that evaluates the ability to quickly change directions while maintaining balance without a loss in speed (Pauole et al., 2000; Chaabene et al., 2018a). The modified COD T-test was performed as previously described by Mhenni et al. (2017, 2021).

### 2.6 Linear Sprint Tests

Athletes’ linear sprint-time was assessed over 5-, 10-, 20- and 30-m intervals as previously described by Chaabene et al. (2018a) and Makhlouf et al. (2022).

### 2.7 Muscular Power Tests

Proxies of muscular power were assessed using vertical jump tests without arm swing (squat and countermovement jumps) and horizontal jump tests to reach the maximal horizontal distance (single-leg hop, single-leg triple hop and 5-jump tests).

The squat and countermovement jumps were assessed with an infrared jump system (Optojump Next instrument, Version 1.3.20.0, Microgate, Bolzano, Italy) according to the procedures described by Tayech et al. (2020).

For the single-leg hop test, participants were instructed to stand on the dominant leg (the kicking leg was identified as the dominant leg) with the toes positioned right behind a mark on the floor. The subjects were then instructed to hop forward as far as possible and to land on the same leg. Participants were allowed to swing the arms freely as they jumped. The horizontal distance was measured from the toe at starting position to the heel where the participant landed. A hop was only regarded as successful if participants were able to keep the foot in place while balancing on one leg until an assessor had marked the landing position (Augustsson et al., 2006; Souissi et al., 2020).

The single-leg triple hop test was performed in the same way as the single-leg hop test with the only difference being that the participant performed three consecutive maximal hops forward on the same leg (dominant leg) to reach the maximal horizontal distance (Makhlouf et al., 2018).

The 5-jump test consists of five consecutive strides in bipedal stance at the start and end of the jumps. At the starting line, participants were not allowed to perform any back step. The athletes were asked to directly jump to the front with the leg of their choice. After the first four strides (i.e., alternating left and right feet for two times each), the athletes had to perform the last stride and end the test again in bipedal stance. If the athletes fell back after completion of the last stride, the test had to be repeated (only two cases of this happened in this study). The 5-jump test was measured from the front edge of the athlete’s feet at the starting position to the heel position after landing. The assessors had to focus on the last stride of the participant in order to exactly determine the last foot print on the mat (Chamari et al., 2008).

The covered distance of the horizontal jump test was recorded by means of a measuring tape to the nearest 1 cm. For each muscular power test, three trials were performed with approximately 2-min passive recovery and the best result was used for further analysis.

### 2.8 Balance Tests

Static balance was assessed utilizing the standing stork balance test and dynamic balance was tested using the Y-balance test (composite score) as described by Chaabene et al. (2018a) and Makhlouf et al. (2022). For each balance test, participants stood on the dominant leg. The best measure was taken from three trials with a 3-min rest in-between.

### 2.9 Statistical Analyses

Two statistical software packages, SPSS 20 (for Windows, Inc., Chicago, IL, United States) and MedCalc (Version 14.8-1993-2014 MedCalc Software) were used for data analyses. Data were presented as means and standard deviations (mean ± SD). The normality of the data distribution was checked using the Shapiro-Wilk test, while homogeneity of variance was assessed by Levene’s test. An independent Student t-test was used to compare performances of top-elite versus elite subgroups, by using the spreadsheet of Hopkins (2007). Given the small sample size (10 males and 10 females) and in order to address the heteroscedasticity problem, the analyses of the performance measures reliability, in the two groups (male and female) separately, were conducted after log transformation (Hopkins, 2000; Hopkins and Gale, 2020), by using the spreadsheet of Hopkins (2015). To determine the relative reliability between the test and retest, the ICC was used. Thresholds for interpreting magnitudes of ICCs as being extremely high, very high, high, moderate, and low were, respectively, 0.99, 0.90, 0.75, 0.50, and 0.20 (Smith and Hopkins, 2011; Hopkins, 2015). Absolute reliability was analyzed by calculating the typical error of measurement (TEM) which was expressed as percentage of the coefficient of variation (CV) (Hopkins, 2000; Chaabene et al., 2018a). The SIC was assumed by multiplying the between-subject SD by 0.2 (SIC_0.2_) which is the typical small effect (Hopkins et al., 2009). The usefulness of each test was assessed by comparing the SIC score with the TEM (Hopkins, 2000). The ability of the test to detect a change was rated as good, ok, or marginal when the TEM was below, similar, or higher than the SIC, respectively. Convergent validity of the TST was established by assessing the relation between TST and other generic performance tests (modified COD T-test, 5-, 10-, 20-, and 30-m linear sprint tests, squat and countermovement jump tests, single-leg hop, single-leg triple hop, and 5-jump tests, standing stork balance and Y-balance tests) using Pearson’s product moment correlation coefficient (*r*) for males and females, separately. The following criteria were adopted to interpret the magnitude of the correlation: trivial (*r* < 0.1), small (0.1 ≤ *r* < 0.3), moderate (0.3 ≤ *r* < 0.5), large (0.5 ≤ *r* < 0.7), very large (0.7 ≤ *r* < 0.9), and nearly perfect (0.9 ≤ *r* ≤ 1) (Hopkins, 2016). Discriminant validity of the TST was analyzed using the receiver operator characteristics (ROC) curve by analyzing the area under the curve (AUC) (Tayech et al., 2020; Makhlouf et al., 2022). The retest outcomes were used to assess the discriminant validity. The ROC curve analysis determined the sensitivity and specificity of a tool to evaluate the ability of the different tests that can discriminate between athletes of different competitive levels (i.e., top-elite vs. elite). The cut-off value for a good discriminative ability was 0.70. Additionally, magnitude-based effect sizes with 90% confidence intervals (CI) were calculated to establish differences between the groups (i.e., top-elite vs. elite), as well as between test and retest (i.e., relative reliability) using the following criteria: ≤0.2 = trivial, >0.2 to 0.6 = small, >0.6 to 1.2 = moderate, >1.2 to 2.0 = large, >2.0 to 4.0 = very large, and >4.0 nearly perfect (Hopkins, 2016).

## 3 Results

The relative and absolute reliability analyses of the TST performance for males and females are displayed in Table 1.

**TABLE 1:** Sex-specific data of taekwondo athletes (means ± standard deviations) computed with log transformed data of reliability statistics for the taekwondo specific change-of-direction speed test with striking (TST) performance.

		TST (s)	Percent Change in Mean and 90% CL	Effect size	ICC	TEM (90% CL) (%)	SIC0.2 (90% CL) (%)
		Mean ± SD		Magnitude (90% CI)	Magnitude (90% CL)		
TST-TO	Male	8.92 ± 0.97	1.1 (−2.4 to 4.7)	0.10	0.86	4.4 (3.2 to 7.3)	1.9 (0.5 to 2.6)
				Trivial (−0.94 to 1.14)	High (0.62 to 0.95)		
	Female	10.09 ± 0.85	−0.7 (−3.0 to 1.7)	−0.07	0.92	2.9 (2.1 to 4.9)	1.7 (0.6 to 2.3)
				Trivial (−1.11 to 0.97)	Very high (0.76 to 0.97)		
TST-TSP	Male	10.57 ± 1.08	−0.4 (−4.0 to 3.3)	−0.06	0.80	4.6 (3.4 to 7.7)	1.6 (-0.1 to 2.2)
				Trivial (−1.10 to 0.98)	High (0.47 to 0.93)		
	Female	11.74 ± 1.34	−1.2 (−5.3 to 3.1)	−0.13	0.80	5.4 (3.9 to 9.0)	1.8 (0.0 to 2.6)
				Trivial (−1.17 to 0.91)	High (0.47 to 0.93)		

TST: Taekwondo Specific Change-of-Direction Speed Test with Striking Techniques; TST-TO: TST-time-only; TST-TSP: TST-time with striking penalty; SD: standard deviation; 90% CI: 90% compatibility interval; 90% CL: 90% compatibility limits; ICC: intraclass correlation coefficient; TEM: Typical error of measurement as a CV (%); SIC_0.2_: Smallest important change based on 0.2 of the between-athlete SD; times/divide factor: ×/÷1.1.

TST outcomes were not different between test and retest, and the estimated effect sizes were trivial in the two groups (Table 1). The results suggest that high relative reliabilities were observed for the TST performances, among males. Whereas for females, high to very high relative reliabilities were observed.

For the absolute reliability, the TEM as a CV (%) for the TST outcomes were relatively low (<5%) in the two groups, except TST -TSP among females, which is slightly higher than 5%. The TEMs exceeded the SICs_0.2_ in the two groups (Table 1).

The correlation coefficient, magnitude, and 90% compatibility limits between athletes’ performance recorded during the TST and their performance during other generic performance tests measuring COD speed, balance, speed and jump performance are summarized in Table 2.

**TABLE 2:** Correlation of log transformed performance variables between TST and generic performance tests measuring COD speed, balance, speed and jump performance in males and females.

		TST performance					
		TST-TO (s)			TST-TSP (s)		
		*r*	Magnitude	90% CL	*r*	Magnitude	90% CL
Modified COD T-test	Male	0.57	Large	(0.03 to 0.86)	0.72	Very large	(0.27 to 0.91)
	Female	0.60	Large	(0.06 to 0.86)	0.59	Large	(0.05 to 0.86)
5-m sprint	Male	0.19	Small	(−0.40 to 0.67)	0.27	Small	(−0.33 to 0.72)
	Female	0.85	Very large	(0.56 to 0.95)	0.74	Very large	(0.32 to 0.92)
10-m sprint	Male	0.23	Small	(−0.37 to 0.69)	0.33	Moderate	(−0.27 to 0.75)
	Female	0.54	Large	(−0.02 to 0.84)	0.62	Large	(0.10 to 0.87)
20-m sprint	Male	0.17	Small	(−0.42 to 0.66)	0.37	Moderate	(−0.23 to 0.76)
	Female	0.71	Very large	(0.26 to 0.91)	0.77	Very large	(0.38 to 0.93)
30-m sprint	Male	0.34	Moderate	(−0.26 to 0.75)	0.51	Large	(−0.05 to 0.83)
	Female	0.77	Very large	(0.38 to 0.93)	0.81	Very large	(0.46 to 0.94)
Squat jump test	Male	−0.47	Moderate	(−0.83 to 0.16)	−0.61	Large	(−0.88 to −0.04)
	Female	−0.35	Moderate	(−0.76 to 0.25)	−0.53	Large	(−0.84 to 0.03)
Countermovement jump test	Male	−0.40	Moderate	(−0.80 to 0.25)	−0.65	Large	(−0.89 to −0.10)
	Female	−0.21	Small	(−0.68 to 0.39)	−0.50	Large	(−0.82 to 0.08)
Single-leg hop test	Male	−0.53	Large	(−0.84 to 0.03)	−0.65	Large	(−0.89 to −0.16)
	Female	−0.92	Nearly perfect	(−0.98 to −0.75)	−0.87	Very large	(−0.96 to −0.61)
Single-leg triple hop test	Male	−0.37	Moderate	(−0.76 to 0.23)	−0.55	Large	(−0.84 to 0.01)
	Female	0.19	Small	(−0.41 to 0.67)	0.25	Small	(−0.35 to 0.70)
5-jump test	Male	−0.34	Moderate	(−0.75 to 0.26)	−0.45	Moderate	(−0.80 to 0.14)
	Female	0.42	Moderate	(−0.17 to 0.79)	0.31	Moderate	(−0.29 to 0.74)
Standing stork balance test	Male	−0.52	Large	(−0.83 to 0.04)	−0.42	Moderate	(−0.79 to 0.17)
	Female	−0.44	Moderate	(−0.80 to 0.14)	−0.39	Moderate	(−0.77 to 0.21)
Y-balance test composite score	Male	−0.64	Large	(−0.88 to −0.14)	−0.74	Very large	(−0.92 to 0.31)
	Female	−0.71	Very large	(−0.91 to −0.25)	−0.55	Large	(−0.84 to 0.01)

TST: Taekwondo Specific Change-of-Direction Speed Test with Striking Techniques; TST-TO: TST-time-only; TST-TSP: TST-time with striking penalty; r: Pearson correlation coefficient; 90% CL: 90% compatibility limits.

For males, moderate-to-large correlations were found between the TST-TO and the modified COD T-test, 30-m sprint test, jump and balance tests. Small correlations were found between the TST-TO and the 5, 10, and 20-m linear sprint tests. Moderate to very large correlations were found between the TST-TSP and the modified COD T-test, and 10, 20, 30-m linear sprint as well as, jump and balance tests. A small correlation was found between the TST-TSP and the 5-m linear sprint test. The highest correlations were observed between the TST outcomes and the Y-balance test (*r* = −0.74), modified COD T-test (*r* = 0.72), and the single-leg hop test (*r* = −0.65).

For females, large to nearly perfect correlations were observed between the TST-TO and the modified COD T-test, linear sprint tests, single-leg hop test, and Y-balance test. Moderate correlations were found between the TST-TO and the squat jump test, 5-jump test, and the stork balance test. Small correlations were observed between the TST-TO and the countermovement jump test, and the single-leg triple hop test. Moderate to very large correlations were found between the TST-TSP and generic performance tests measuring COD speed, balance, speed, and jump performance, except for the single-leg triple hop test, which showed a small correlation with TST-TSP. The highest correlations were found between the TST test and the single-leg hop test (*r* = −0.92), 5-m (*r* = 0.85), 30-m sprint test (*r* = 0.81), and the Y-balance test (*r* = −0.71).

Group-specific data (top-elite vs. elite taekwondo athletes) regarding the TST and TECHN-INDEX are displayed in Table 3. Regardless of sex, top-elite compared with elite athletes showed better performances in TST together with a lower TECHN-INDEX.

**TABLE 3:** Comparison of TST performance and TECHN-INDEX between top-elite and elite taekwondo athletes.

	Top-elite (*n* = 10)	Elite (*n* = 10)	Mean difference (90% CL) (s)	Effect size
	Mean ± SD (s)	Mean ± SD (s)		Magnitude (90% CI)
TST-TO	8.89 ± 0.72	10.15 ± 0.86	−1.26 (0.65 to 1.88)	1.60
				Large (0.75 to 2.43)
TST-TSP	10.29 ± 0.63	11.80 ± 0.88	−1.51 (0.92 to 2.11)	1.97
				Large (1.08 to 2.87)
TECHN-INDEX	2.14 ± 0.44	2.92 ± 0.78	−0.78 (0.28 to 1.28)	1.23
				Large (0.43 to 2.03)

TST: Taekwondo Specific Change-of-Direction Speed Test with Striking Techniques; TST-TO: TST-time-only; TST-TSP: TST-time with striking penalty; TECHN-INDEX: Technical index (TST-TO minus modified COD T-test); SD: standard deviation; 90% CI: 90% compatibility interval; 90% CL: 90% compatibility limits.

For male athletes, TST and TECHN-INDEX performance were better in top-elite (*n* = 5) versus elite athletes (*n* = 5) [for TST-TO: 8.43 ± 0.72 s vs. 9.58 ± 0.38 s, respectively, with a large effect size (=2.0); for TST-TSP: 9.93 ± 0.68 s vs. 11.08 ± 0.10 s, respectively, with a very large effect size (= 2.4); for TECHN-INDEX: 1.94 ± 0.58 s vs. 2.92 ± 0.47 s, respectively, with a large effect size (=1.9)]. For female athletes, TST and TECHN-INDEX performance were better in top-elite (*n* = 5) compared with elite athletes (*n* = 5) [for TST-TO: 9.34 ± 0.36 s vs. 10.72 ± 0.83 s, respectively, with a very large effect size (=2.2); for TST-TSP: 10.64 ± 0.32 s vs. 12.52 ± 0.65 s, respectively, with a very large effect size (=3.7); for TECHN-INDEX: 2.34 ± 0.06 s vs. 2.92 ± 1.07 s, respectively, with a small effect size (=0.8)]. Notably, top-elite female athletes outperformed male elite athletes in TST and TECHN-INDEX performance [for TST-TO: 9.34 ± 0.36 s vs. 9.58 ± 0.38 s, respectively, with a small effect size (=0.6); for TST-TSP: 10.64 ± 0.32 s vs. 11.08 ± 0.10 s, respectively, with a large effect size (=1.9); for TECHN-INDEX: 2.34 ± 0.06 s vs. 2.92 ± 0.47 s, respectively, with a large effect size (=1.7)].

**FIGURE 2:**
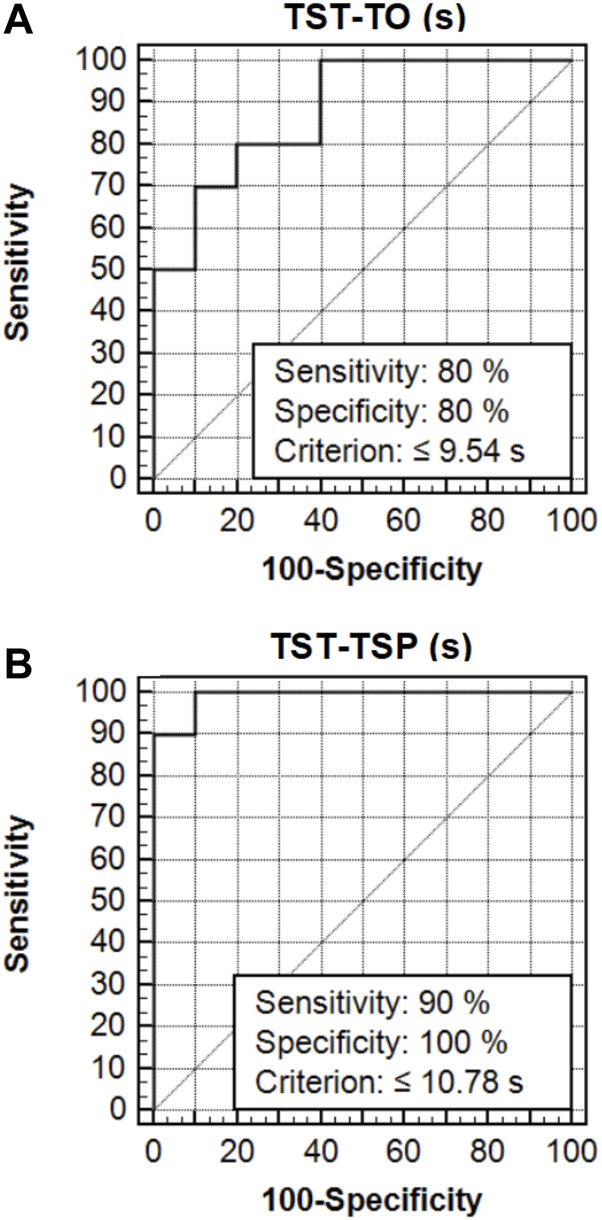
ROC curves for the TST performance for top-elite and elite taekwondo athletes. **(A)**: TST-TO (s); **(B)**: TST-TSP (s); TST: Taekwondo specific change-of-direction speed test with striking techniques; TST-TO: TST-time-only; TST-TSP: TST-time with striking penalty; ROC: Receiver operating characteristics.

The TST was considered to have very good discriminant validity. The area under the ROC curve (AUC) was 0.88 (standard error = 0.075; 95% CI: 0.66–0.98) for TST-TO (Figure 2A), 0.99 (standard error = 0.014; 95% CI: 0.81–1.00) for TST-TSP (s) (Figure 2B). The cut-off performances for discriminating between the top-elite and elite athletes were ≤9.54 s [sensitivity of 80% (95% CI = 44.4 to 97.5) and specificity of 80% (95% CI = 44.4 to 97.5)] for TST-TO (s) (Figure 2A), ≤10.78 s [sensitivity of 90% (95% CI = 55.5 to 99.7) and specificity of 100% (95% CI = 69.2 to 100)] for TST-TSP (s) (Figure 2B).

## 4 Discussion

The main findings of this study demonstrated 1) high relative and acceptable absolute reliability in TST performances in both, males and females; 2) high associations of the TST with the modified COD T-test, the Y-balance test, and the single-leg hop test in males; 3) high associations of the TST with the 5-, 20- and 30-m linear sprint tests, single-leg hop test, and Y-balance test in females; and 4) very good discriminatory capability between top-elite and elite athletes, regardless of sex.

Previous studies conducted with combat sports COD speed tests have found an ICC of 0.97, among fencing athletes, when performing a new specific fencing COD test (Chtara et al., 2020), and an ICC of 0.97, among taekwondo athletes, when performing the Taekwondo-specific agility test (Chaabene et al., 2018a). Our results showed that ICC values ranged from 0.80 to 0.92, thus indicating high to very high relative reliability. Brughelli et al. (2008) reported that all the tests that have been used to measure COD ability show similar reliability (ICC ranged from 0.80 to 0.96), regardless of the duration of the test, the number of CODs, or the direction in which most of the forces were applied.

In summary, this study revealed that the TEMs (expressed as CV) relating to TST performances were equal to or less than the 5% limit and exceeded the SIC_0.2_ in both groups. Comparable results were reported by Chaabene et al. (2018a) and Chtara et al. (2020). Thereby, TST presented high relative and acceptable absolute reliability, with a more precise measure. In view of the above results, we suggest that this tool, that needs specific equipment [electronic protection devices (i.e., body, headgear protectors and hand–foot protectors)] available to many coaches and athletes, presents good specificity and feasibility, which represent an important aspect in performance testing for striking combat sports (Chaabene et al., 2018b; Tayech et al., 2019, 2020, 2022; Ribeiro et al., 2020).

The test-retest reliability depends on a number of factors such as the number of participants, number of performed trials, participant’s skill level, homogeneity of the sample, environmental conditions, time of the day of the test’s administration (Chaabene et al., 2018b; Kerdaoui et al., 2021; Makhlouf et al., 2022), and most importantly the adequate precision of estimates of change in the variable of the experimental study (Hopkins, 2000). About this, Hopkins (2000) explained that the paramount concern in the design of any study is adequate precision for the estimates of the outcome measures. The author goes on to say that in a reliability study, the most important outcome measures are the TEM and the change in the mean between trials. The present study included twenty elite taekwondo athletes including 10 males and 10 females. Although this number is less than the participants (n ranged from 27 to 39) in previous similar combat sports studies, we observed high relative and acceptable absolute reliability (Chaabene et al., 2018a; Chtara et al., 2020), which can be explained by the participants’ test familiarization, and the number of trials (i.e., three trials) performed during each session (i.e., test and retest). Moreover, the participants belonged to a homogeneous group, were highly motivated, had the same training schedules, and trained together under the same coaches. Finally, the experiments were carried out in almost the same environmental conditions, and at the same time of the day.

The COD T-test and/or modified COD T-test have been used in previous studies to determine the criterion and/or convergent validity of other generic COD speed tests (Pauole et al., 2000; Sassi et al., 2009; Chaabene et al., 2018a; Chtara et al., 2020). Nimphius et al. (2018) have reported that T-test could allow for more complex skills valid assessment of COD speed (e.g., due to modes and number of changes in direction). In addition, Sassi et al. (2009) indicated that the modified COD T-test, as well as the COD T-test, could be used to assess COD speed, and hence planned agility. In the current study, the modified COD T-test was selected as the convergent measure for TST validation, because it purportedly measures quickness when changing direction over a relatively short distance (Pauole et al., 2000; Sassi et al., 2009; Chaabene et al., 2018a, 2020), and represents the most used COD speed test in taekwondo (Marković et al., 2005; Monks et al., 2017; Chaabene et al., 2018a, 2020; Taskin and Akkoyunlu, 2020). Additionally, consistent with the analysis described by Nimphius et al. (2018) regarding the relevant techniques of COD speed tests, the TST included different trunk position and control, orientation of the hips relative to the intended direction of shifts and striking techniques, rear or front foot-strike during the stance phase, knee flexion during braking, and arm actions and visual focus especially during the COD and striking techniques on the dummy. In view of the above considerations, and in accordance with the results of Sassi et al. (2009), we observed large to very large correlations between TST performances with the modified COD T-test in both, males (*r* = 0.57 for TST-TO, and *r* = 0.72 for TST-TSP) and females (*r* = 0.60 and 0.37 for TST-TO, and *r* = 0.59 for TST-TSP). When combining all participants (males and females), Pearson’s correlation coefficients revealed very large associations between TST outcomes and performance in the modified COD T-test (*r* = 0.69 for TST-TO, and *r* = 0.71 for TST-TSP); corroborating the findings of Chaabene et al. (2108a), who demonstrated that a taekwondo-specific agility test highly correlated (very large magnitude) with the T-test in male and female elite taekwondo athletes (*r* = 0.71). Recently, similar results were reported by Chtara et al. (2020) who found large to very large correlations between a specific fencing COD test and the T-test, regardless of sex (males: *r* = 0.75; females: *r* = 0.79; combined: *r* = 0.87), in fencing athletes. More recently, Makhlouf et al. (2022) reported that a new soccer-specific COD speed test (Illinois COD test with ball dribbling speed) correlated with a large magnitude with the Illinois COD test without the ball in young soccer players of different biological maturity and playing levels (*r* = 0.65). These findings support the convergent validity of the TST among young taekwondo athletes.

Our results showed a moderate relation (*r* = 0.34) between the TST-TO and the 30-m linear sprint test in males. Associations between TST-TO and the 5-, 10-, and 20-m linear sprint tests were small (*r* = 0.17 to 0.23). In females, the correlations between TST-TO and the linear sprint tests were mostly very large (*r* = 0.71 to 0.85). Regarding TST performance with penalty for striking techniques, the correlations between TST-TSP and linear sprint tests, were moderate with 10-m and 20-m tests (*r* = 0.33 and 0.37, respectively), and large with 30-m linear sprints (*r* = 0.51), in males. However, the correlation was small with the 5-m linear sprint test (*r* = 0.27). In females, correlations between the TST-TSP and linear sprint tests were mostly very large (*r* = 0.74 to 0.81). Concordant with our results, Sassi et al. (2009) found that performance in the 10-m linear sprint test did not correlate with the modified COD T-test in male physical education students. However, in females, the authors found a moderate association (*r* = 0.34) between the two tests. Compared to the findings of Sassi et al. (2009), large (*r* = 0.62) and moderate (*r* = 0.33) correlations were found in our study for females and males, respectively. This can be explained by the participants’ skill level, and homogeneity of the sample in our study (Makhlouf et al., 2022). It has previously been reported that correlations between tests are highly influenced by participants’ heterogeneity (Sassi et al., 2009; Chtara et al., 2020). Accordingly, the pooling of all participants in one group increased heterogeneity and concomitantly the respective correlation coefficients (Sassi et al., 2009; Chaabene et al., 2018a; Chtara et al., 2020). These findings concur well with those of Chaabene et al. (2018a), who reported large associations (5-m: *r* = 0.52 and 20-m: *r* = 0.58) between a taekwondo-specific agility test and linear sprint tests in elite taekwondo athletes. Likewise, Arabaci et al. (2010) found large to very large positive relations between a COD speed test (20-m zigzag) and 20- and 30-m linear speed performance in taekwondo athletes (*r* = 0.83 and *r* = 0.61, respectively). This meaningful relation between these two tests has also been found in athletes from different sports. Recently, Makhlouf et al. (2022) found a large correlation between 10- and 30-m linear sprint speed tests and the Illinois COD test with ball dribbling speed in young soccer players of different biological maturity and playing levels (*r* = 0.52 and *r* = 0.59, respectively). Numerous studies have supported the relation between measures of linear sprint speed and COD speed. However, it seems that the magnitude of correlations depends on the distance of the sprint, the type of the COD speed test (Horníková and Zemková, 2021), and the sex (Sassi et al., 2009; Delextrat et al., 2015). Horníková and Zemková, (2021) reported that the type of COD speed test could potentially affect the relationship between COD speed and linear sprint speed; therefore, the strength of correlation may also be dependent on the similarity and/or some degree of compliance between the COD speed test and the specific distance of the linear sprint. On the other hand, several studies have examined the speed characteristics of taekwondo athletes using conventional field-based testing methods, including sprint tests ranging from 5-m to 30-m (Bridge et al., 2014). The 5-m linear sprint test has often been used to assess quickness performance in taekwondo athletes (Chaabene et al., 2018a; Taskin and Akkoyunlu, 2020). The very large association between the TST and the 5-m linear sprint test in female taekwondo athletes revealed that the relative speed of the first acceleration steps appears to be an important component in determining COD speed over short distances (Sayers, 2015). This is in accordance with previous studies that reported acceleration as an important aspect in COD movements (Young et al., 2002; Sheppard and Young, 2006; Zemková and Hamar, 2017; Chaabene et al., 2018a; Chtara et al., 2020). However, the ability to decelerate rapidly during the TST is also a key component of COD ability (Sayers, 2015). It has been reported that when undertaking a 180° COD speed test, deceleration movement times are extremely variable within and between individuals, particularly when compared with acceleration movement times (Sayers, 2015). Accordingly, Zemková and Hamar (2017) reported that the COD speed tests evaluate an athlete’s ability to rapidly decelerate and reaccelerate in the new direction. Mostly, the 5-m linear sprint correlated only with the short COD speed tests, while higher distance in linear speed results showed a stronger correlation with the many type of COD speed tests (Horníková and Zemková, 2021). In this sense, a large positive correlation (*r* = 0.65) was found between the best trial in the modified COD T-test and the 10-m sprint test in elite female soccer players (Lockie et al., 2018). Other studies reported moderate-to-large correlations (*r* = 0.39 to 0.65) between the COD speed test and a 10-m linear sprint test in team sport players. This result is remarkable because the test design is considerably different between the two tests (Lockie et al., 2018; Mackala et al., 2020; Horníková and Zemková, 2021). Beyond a distance of 10-m linear sprint, it has been found that COD speed performance correlated with acceleration speed for longer distances (from 18.3-m to 40-m) in handball and soccer players (*r* = 0.25 to 0.84) (Horníková and Zemková, 2021). Concordantly, our results indicate that TST performances correlate more strongly with maximum running speed than with acceleration speed, mainly in male taekwondo athletes. It has been reported that the correlation coefficients between either maximal sprinting time over distances up to 40-m and the performance of various COD speed tests ranged from large to very large (Salaj and Markovic, 2011).

This study revealed moderate to large negative correlations (*r* = −0.34 to −0.65) between TST outcomes and measures of jump performance in males. In females, the relation between tests were mostly in the range of moderate to large. The highest correlations were found in females between the TST test and the single-leg hop test (nearly perfect for TST-TO and very large for TST-TSP). However, the correlations were small between the TST-TO and the countermovement jump as well as the single-leg triple hop test, and between the TST-TSP and the single-leg triple hop test in females. Previous studies have examined the relation between COD speed tests and the countermovement jump test and have reported controversial results (Peterson et al., 2006; Sassi et al., 2009). Sassi et al. (2009) showed that the modified COD T-test did not correlate with performance in the countermovement jump test in male physical education students. However, the same test correlated moderately with the countermovement jump test in females (*r* = −0.47). Moreover, Peterson et al. (2006) reported a very large correlation between the COD T-test and the countermovement jump test for young female (*r* = −0.71), but not for young male collegiate athletes. These differences between the outcomes of this study and the literature can most likely be explained by several moderating factors such as sex, age, and level of fitness and expertise, the distances and constraints in the tests, and the type of directional change in different COD speed tests. Moreover, the small sample size (i.e., 10 males and 10 females), and heterogeneity of participants in both groups (combining top- and elite athletes) might have affected the association between the TST and the respective jump tests.

Regardless of sex, this study revealed large to very large negative relations between TST outcomes and jump tests. These findings are concordant and/or even better than the findings of the previous scientific literature in taekwondo highlighting moderate to large correlations between the taekwondo specific COD speed test (i.e., Taekwondo-specific agility test) and vertical (i.e., squat and countermovement jumps) and horizontal (i.e., standing long jump and single-leg triple hop) jumping tests in elite taekwondo athletes (Chaabene et al., 2018a). The literature showed a strong association between COD speed and vertical (*r* = −0.68 to −0.77) and horizontal (*r* = −0.56 to −0.65) jump performances (McFarland et al., 2016; Horníková and Zemková, 2021; Makhlouf et al., 2022). Moreover, Young et al. (2002) reported that leg muscle power is important determinant of COD ability. In accordance with our results, McFarland et al. (2016) found that squat and countermovement jumps were correlated to the T-test for elite women soccer players (*r* = −0.68 and *r* = −0.76, respectively). In addition, it has been documented that the countermovement jump height correlated with the COD speed tests in almost all studies in female (*r* = −0.39 to −0.79) or mixed group of male and female players (*r* = 0.56 and *r* = 0.84, respectively) (Horníková and Zemková, 2021). Peterson et al. (2006) reported greater correlations for horizontal compared with vertical jump tests and COD speed tests. In addition, different studies revealed that performance in unipedal compared with bipedal jumps might be closer related with participants’ COD speed (Young et al., 2002; Delextrat et al., 2015). In this regard, different studies confirmed the strong relation between the single-leg hop test and COD speed tests with lateral running or turning 180°, like the COD T-test (Horníková and Zemková, 2021). In agreement with our results, Negrete and Brophy (2000) found a large negative correlation (*r* = −0.65) between the single-leg hop test and a diamond-shaped agility test. In view of the above considerations, it has been reported that if the jump test closely mimics the respective sport-specific movement, the relation with COD speed performance is higher (Horníková and Zemková, 2021). Concordantly, this study showed that TST performance strongly correlates with the single-leg hop test but to a lesser degree with the squat jump, countermovement jump, single-leg triple hop, and the 5-jump test, primarily in female taekwondo athletes. This might be explained by the fact that during taekwondo combat, the exchanges of kicks between the opponents are done unilaterally and mostly following a unipedal horizontal jump, especially when executing the block technique (in taekwondo terminology referred to “Yop-chagi”) with the front leg. It has been reported that COD tests require a braking force followed by a propulsive force, which in turn may increase the importance of eccentric-concentric force capability of leg muscles as during horizontal jumps (Brughelli et al., 2008). This is how Brughelli et al. (2008) claimed that jumps that involve the combination of both horizontal and vertical ground reaction forces may better predict COD ability. Based on the current study’s results and consistent with the findings of Pauole et al. (2000), although the TST is a reliable and valid test to measure the taekwondo specific COD speed combined to striking techniques, it is not surprising that leg speed and power contributes substantially to the variability in TST performance. Against this background, it has been well documented that speed and power are essential elements in successful scoring in taekwondo sparring. Most importantly, with the introduction of the PSS, displacement and attack speeds became extremely important in the combat as without a high speed, an opponent could block the attack or dodge away from the attack easily. In addition, failing to touch the opponent’s body sensor with the athlete’s own foot sensor and with an optimal power would result in zero score (Kwok and Cheung, 2021). Therefore, since TST involve decelerations, re-accelerations and constant adjustments of steps and body posture mainly during the COD actions and unilateral kicking actions, the potential to improve COD speed and striking power by linear sprint and jump training (mainly unipedal horizontal jump) is recommended. In view of the above considerations, future research should examine the relationship of TST performance to various taekwondo-specific performance measures.

There were moderate to very large associations between measures of TST and variables of static and dynamic balance in both groups. The highest correlations (*r* = −0.55 to −0.74) were found between TST outcomes and measures of dynamic balance in both groups. This result is in agreement with findings from Chaabene et al. (2018a), who observed a large negative relation (*r* = −0.59) between the taekwondo-specific agility test and the Y-balance test in elite taekwondo athletes. Taekwondo performance appears to be related with measures of COD speed, muscular power, as well as static and dynamic balance, because during a combat, athletes require rapid and powerful defense and attack from all directions using both sides of his/her body (Fong et al., 2012; Negahban et al., 2013). It has been reported that during taekwondo fights elite athletes could turn and kick at high speeds (5.2 to 16.26 m·s^−1^) and generate huge amount of striking forces (390.7 to 661.9 N) without losing balance (Fong et al., 2012). In addition to these turning and kicking abilities, taekwondo fighters adjust their body position and distance to the opponent frequently by stepping to different directions (i.e., COD) in order to score points. As in TST, commonly used unipedal standing and jumping kick techniques in taekwondo combat include roundhouse kick, side kick and back kick. Indeed, rotation of the body’s fighter and pivoting on one leg is an essential component in all of these kicking skills (Fong et al., 2012). Besides, taekwondo athlete requires good static and dynamic balance not only to optimize their kicks, but also to prevent falls and to prevent a penalty from the referee (in taekwondo terminology referred to “Gam-Jeom”), as well as avoid the dangerous situation of being re-attacked by his or her opponent (Menescardi et al., 2019). Therefore, postural control is crucial for taekwondo athletes due to its dynamic kicking nature and the requirements of the game rules during combats (Fong et al., 2012).

In this study, correlations between TST and proxies of athletic performance (i.e., modified COD T-test, linear sprint and muscular power) were higher when striking techniques were counted as a performance score in TST (i.e., TST-TSP), in both groups. This might be explained by the fact that, as during taekwondo matches, in TST, the implementation of the PSS ensure that athletes would receive a score only if they could kick the body sensor of the opponent (i.e., dummy) with the foot sensor of their own with sufficient level of impact (i.e., power). These taekwondo electronic protection devices (i.e., body, headgear and hand–foot protectors) have a technical feature that automatically recognizes the effective attack power by means of a sensor equipped with an advanced electronic chip attached to the protector, which automatically transmits it to the score monitor through a wireless transmission device (Sevinç, 2017; World Taekwondo, 2020; Park et al., 2021, Tayech et al., 2019, 2020, 2022). Therefore, the use of PSS in TST was a measurement to ensure a reliable and accurate score identification during the test. On the other hand, and based on the current study’s findings, we can suggest that during the TST, the fighter adopts both offensive and defensive combat styles as described by Menescardi et al. (2019). The offensive style implies that the fighter quickly moves forward, invading the space of the attacker, and competing over a shorter distance by responding to attacks with counterattacks (i.e., punch as during TST). While the defensive style implies that the fighter makes dodges and speedy COD while employing striking defensive technics (i.e., blocks and kicks as during TST) (Menescardi et al., 2019; Kwok and Cheung, 2021). Hence, instead of limiting on one kicking technique (i.e., roundhouse kick) as in Taekwondo-specific agility test (Chaabene et al., 2018a), coaches should include different types of kicking and punch techniques in the training, so athletes would utilize these skills as in TST (Kwok and Cheung, 2021).

One of the most pertinent aims of this study was to assess TST performance with the goal to discriminate between taekwondo athletes of different expertise levels (top-elite vs. elite athletes). We found better TST performance in top-elite compared to elite taekwondo athletes. Another noteworthy result of this study was that mean TST performance was better in top-elite female versus male elite athletes. It has been well-documented that a ROC analysis is an appropriate statistical approach to examine the discriminative power and the responsiveness of performance tests (Impellizzeri and Marcora, 2009; Chaabene et al., 2018b). In view of the above consideration, we applied a ROC analysis and the findings showed a very good discriminative ability of the TST, irrespective of sex (de Vet et al., 2001; Mannion et al., 2005; Chaabene et al., 2018a, 2018b; Tayech et al., 2020, 2022; Makhlouf et al., 2022). However, a single cut-off score to discriminate top-elite from elite athletes without taking sex into consideration appears not to be adequate (Chaabene et al., 2018a; Chtara et al., 2020; Tayech et al., 2020, 2022). Therefore, these TST scores could penalize female athletes. Larger sample sizes including males and females would most likely reveal sex-specific discrimination cut-offs for the TST scores (Evans et al., 2021). COD speed tests have been used in several sports to discriminate top-elite from elite taekwondo athletes (Chaabene et al., 2018a), high- from low-ranked fencer athletes (Chtara et al., 2020), as well as young soccer players of different biological maturity and playing levels (Makhlouf et al., 2022), thus representing a practically relevant tool for talent identification and selection (Chaabene et al., 2020). The findings of this study are similar to those of Chaabene et al. (2018a), where top-elite athletes outperformed elite taekwondo athletes in specific COD speed measures. Of interest, in the present study, when the different striking techniques were counted in the test performance, the discriminative ability of TST became more important. This may also be justified by the better TECHN-INDEX scores (Hachana et al., 2014; Fiorilli et al., 2017; Conte et al., 2020) in top-elite compared to elite taekwondo athletes. This last finding could highlight that better technical skills may also be determinants of success (Makhlouf et al., 2022) among top-elite compared to elite taekwondo athletes. Based on the above results, coaches could use the TST as an appropriate tool to distinguish between taekwondo athletes of various competitive levels.

### 4.1 Strengths and Limitations

As with any scientific study, the current study includes some limitations that must be acknowledged. First, the sample size is small and included two levels of competitive athletes only. Therefore, further investigations should check the reliability and validity of the TST in larger number of taekwondo athletes of different ages, sex, and athletic levels. Second, the ability to COD in response to a stimulus that cannot be preplanned was not assessed in this study. Given that taekwondo is an open skill sport, athletes need to adjust their movements quickly in response to the dynamic situation (i.e., opponent’s actions) during combats. Accordingly, the use of specific reactive agility tests that combine COD and/or speed with cognitive measures is needed in future investigations (Sheppard and Young, 2006; Zemková and Hamar, 2013, 2014, 2017). Despite these limitations, the present findings provide a valuable opportunity for the assessment of specific COD speed combined to striking techniques counted through the taekwondo electronic scoring system. Further research is needed to discern the sensitivity of this new test throughout different phases of the competitive season to detect small changes in performance among taekwondo athletes.

## 5 Conclusion

The TST has shown good validity in elite taekwondo athletes, and can effectively discriminate taekwondo athletes of different expertise level. Although the usefulness of the TST is questioned to detect small performance changes in the present population, the TST can detect moderate changes in taekwondo-specific COD speed. Moreover, as measures of COD speed, linear sprint time, muscle power, and static and dynamic balance are highly associated with TST, it is recommended that all-out exercises and static-dynamic balance exercises should be included to develop COD speed together with taekwondo specific skills in taekwondo athletes. Therefore, our results support the use of the TST to assess taekwondo athletes’ COD speed with striking ability. The TST test is a simple and practical tool for coaches, and does not require invasive equipment. Moreover, the current test is appropriate to investigate the differences between performance according to the expertise level (top-elite versus elite). In addition, this test has potential training applications, because the test can be used as a practical COD and power-training exercise using widely applied striking techniques during taekwondo combat.

## Data Availability

The raw data supporting the conclusion of this article will be made available by the authors, without undue reservation.

## References

[B1] Arabaci R. Görgülü R. Çatikkaş F. (2010). Relationship between Agility and Speed, Reaction Time and Body Mass index in Taekwondo Athletes. Sport Sci. 5, 71–77.

[B2] Augustsson J. Thomee R. Linden C. Folkesson M. Tranberg R. Karlsson J. (2006). Single-leg Hop Testing Following Fatiguing Exercise: Reliability and Biomechanical Analysis. Scand. J. Med. Sci. Sports 16, 111–120. 10.1111/j.1600-0838.2005.00446.x 16533349

[B3] Bouhlel E. Jouini A. Gmada N. Nefzi A. Ben Abdallah K. Tabka Z. (2006). Heart Rate and Blood Lactate Responses during Taekwondo Training and Competition. Sci. Sports 21, 285–290. 10.1016/j.scispo.2006.08.003

[B4] Bridge C. A. Ferreira da Silva Santos J. Chaabène H. Pieter W. Franchini E. (2014). Physical and Physiological Profiles of Taekwondo Athletes. Sports Med. 44, 713–733. 10.1007/s40279-014-0159-9 24549477

[B5] Brughelli M. Cronin J. Levin G. Chaouachi A. (2008). Understanding Change of Direction Ability in Sport. Sports Med. 38, 1045–1063. 10.2165/00007256-200838120-00007 19026020

[B6] Chaabene H. Negra Y. Bouguezzi R. Capranica L. Franchini E. Prieske O. (2018b). Tests for the Assessment of Sport-specific Performance in Olympic Combat Sports: a Systematic Review with Practical Recommendations. Front. Physiol. 9, 386. 10.3389/fphys.2018.00386 29692739 PMC5902544

[B7] Chaabene H. Negra Y. Capranica L. Bouguezzi R. Hachana Y. Rouahi M. A. (2018a). Validity and Reliability of a New Test of Planned Agility in Elite Taekwondo Athletes. J. Strength Cond. Res. 32, 2542–2547. 10.1519/JSC.0000000000002325 29120989

[B8] Chaabene H. Prieske O. Moran J. Negra Y. Attia A. Granacher U. (2020). Effects of Resistance Training on Change-Of-Direction Speed in Youth and Young Physically Active and Athletic Adults: a Systematic Review with Meta-Analysis. Sports Med. 50, 1483–1499. 10.1007/s40279-020-01293-w 32451922 PMC7376516

[B9] Chamari K. Chaouachi A. Hambli M. Kaouech F. Wisløff U. Castagna C. (2008). The Five-Jump Test for Distance as a Field Test to Assess Lower Limb Explosive Power in Soccer Players. J. Strength Cond. Res. 22, 944–950. 10.1007/s40279-020-01293-w10.1519/jsc.0b013e31816a57c6 18438217

[B10] Cho E.-H. Eom H.-J. Jang S.-Y. (2020). Comparison of Patterns of Skill Actions between Analog and Electronic Protectors in Taekwondo: A Log-Linear Analysis. Ijerph 17, 3927. 10.3390/ijerph17113927 PMC7312946 32492955

[B11] Chtara H. Negra Y. Chaabene H. Chtara M. Cronin J. Chaouachi A. (2020). Validity and Reliability of a New Test of Change of Direction in Fencing Athletes. Ijerph 17, 4545. 10.3390/ijerph17124545 PMC7345253 32599790

[B12] Conte D. Scanlan A. T. Dalbo V. Gang S. Smith M. Bietkis T. (2020). Dribble Deficit Quantifies Dribbling Speed Independently of Sprinting Speed and Differentiates between Age Categories in Pre-adolescent Basketball Players. bs 37, 261–267. 10.5114/biolsport.2020.95637 PMC7433332 32879548

[B13] de Vet H. C. W. Bouter L. M. Bezemer P. D. Beurskens A. J. H. M. (2001). Reproducibility and Responsiveness of Evaluative Outcome Measures. Int. J. Technol. Assess. Health Care 17, 479–487. 10.1017/s0266462301107038 11758292

[B14] Del Vecchio F. B. Franchini E. Del Vecchio A. H. M. Pieter W. (2011). Energy Absorbed by Electronic Body Protectors from Kicks in a Taekwondo Competition. Biol. Sport 28, 75–78. 10.5604/935878

[B15] Delextrat A. Grosgeorge B. Bieuzen F. (2015). Determinants of Performance in a New Test of Planned Agility for Young Elite Basketball Players. Int. J. Sports Physiol. Perform. 10, 160–165. 10.1123/ijspp.2014-0097 24956606

[B16] Evans N. A. Konz S. Nitz A. Uhl T. L. (2021). Reproducibility and Discriminant Validity of the Posterior Shoulder Endurance Test in Healthy and Painful Populations. Phys. Ther. Sport 47, 66–71. 10.1016/j.ptsp.2020.10.014 33197875

[B17] Farhani F. Rajabi H. Negaresh R. Ali A. Amani Shalamzari S. Baker J. S. (2019). Reliability and Validity of a Novel Futsal Special Performance Test Designed to Measure Skills and Anaerobic Performance. Int. J. Sports Physiol. Perform. 14, 1096–1102. 10.1123/ijspp.2018-0850 30702380

[B18] Fiorilli G. Iuliano E. Mitrotasios M. Pistone E. M. Aquino G. Calcagno G. (2017). Are Change of Direction Speed and Reactive Agility Useful for Determining the Optimal Field Position for Young Soccer Players? J. Sports Sci. Med. 16, 247–253. 28630578 PMC5465987

[B19] Fong S. S. M. Cheung C. K. Y. Ip J. Y. Chiu J. H. N. Lam K. L. H. Tsang W. W. N. (2012). Sport-specific Balance Ability in Taekwondo Practitioners. Jhse 7, 520–526. 10.4100/jhse.2012.72.15

[B20] Gaamouri N. Zouhal H. Hammami M. Hackney A. C. Abderrahman A. B. Saeidi A. (2019). Effects of Polyphenol (Carob) Supplementation on Body Composition and Aerobic Capacity in Taekwondo Athletes. Physiol. Behav. 205, 22–28. 10.1016/j.physbeh.2019.03.003 30853622

[B21] Guimarães A. N. Ugrinowitsch H. Dascal J. B. Okazaki V. H. A. (2020). Controlling Degrees of Freedom in Learning a Taekwondo Kick. Mot. Control. 24, 512–526. 10.1123/mc.2019-0118 32732451

[B22] Hachana Y. Chaabène H. Ben Rajeb G. Khlifa R. Aouadi R. Chamari K. (2014). Validity and Reliability of New Agility Test Among Elite and Subelite under 14-soccer Players. PloS one 9, e95773. 10.1371/journal.pone.0095773 24752193 PMC3994134

[B23] Hopkins W. G. (2016). A Scale of Magnitudes for Effect Statistics. Sport Sci. A New view Stat. 502, 411.

[B24] Hopkins W. G. (2007). A Spreadsheet to Compare Means of Two Groups. Sport Sci. 11, 22–23.

[B25] Hopkins W. G. Marshall S. W. Batterham A. M. Hanin J. (2009). Progressive Statistics for Studies in Sports Medicine and Exercise Science. Med. Sci. Sports Exerc. 41, 3–12. 10.1249/MSS.0b013e31818cb278 19092709

[B26] Hopkins W. G. (2000). Measures of Reliability in Sports Medicine and Science. Sports Med. 30, 1–15. 10.2165/00007256-200030010-00001 10907753

[B27] Hopkins W. G. (2015). Spreadsheets for Analysis of Validity and Reliability. Sport Sci. 19, 36–42.

[B28] Hopkins W. G. (2020). When N Is <10: How to Cope with Very Small Samples. Sport Sci. 24, 1.

[B29] Horníková H. Zemková E. (2021). Relationship between Physical Factors and Change of Direction Speed in Team Sports. Appl. Sci. 11, 655. 10.3390/app11020655

[B30] Impellizzeri F. M. Marcora S. M. (2009). Test Validation in Sport Physiology: Lessons Learned from Clinimetrics. Int. J. Sports Physiol. Perform. 4, 269–277. 10.1123/ijspp.4.2.269 19567929

[B31] Janowski M. Zieliński J. Ciekot-Sołtysiak M. Schneider A. Kusy K. (2020). The Effect of Sports Rules Amendments on Exercise Intensity during Taekwondo-specific Workouts. Ijerph 17, 6779. 10.3390/ijerph17186779 PMC7559273 32957546

[B32] Kerdaoui Z. Sammoud S. Negra Y. Attia A. Hachana Y. (2021). Reliability and Time-Of-Day Effect on Measures of Change of Direction Deficit in Young Healthy Physical Education Students. Chronobiology Int. 38, 103–108. 10.1080/07420528.2020.1839091 33317349

[B33] Kwok H. H. M. Cheung S. Y. (2021). Notational Analysis of Fighting Tactics in Taekwondo Athletes with Three Levels of Expertise. J. Multidiscip. Res. 13, 59–69.

[B34] Lockie R. Dawes J. Jones M. (2018). Relationships between Linear Speed and Lower-Body Power with Change-Of-Direction Speed in National Collegiate Athletic Association Divisions I and II Women Soccer Athletes. Sports 6, 30. 10.3390/sports6020030 PMC6026790 29910334

[B35] Mackala K. Vodičar J. Žvan M. Križaj J. Stodolka J. Rauter S. (2020). Evaluation of the Pre-planned and Non-planed Agility Performance: Comparison between Individual and Team Sports. Ijerph 17, 975. 10.3390/ijerph17030975 PMC7037819 32033236

[B36] Makhlouf I. Chaouachi A. Chaouachi M. Ben Othman A. Granacher U. Behm D. G. (2018). Combination of Agility and Plyometric Training Provides Similar Training Benefits as Combined Balance and Plyometric Training in Young Soccer Players. Front. Physiol. 9, 1611. 10.3389/fphys.2018.01611 30483158 PMC6243212

[B37] Makhlouf I. Tayech A. Arbi Mejri M. Haddad M. G Behm D. Granacher U. (2022). Reliability and Validity of a Modified Illinois Change-Of-Direction Test with ball Dribbling Speed in Young Soccer Players. bs 39, 295–306. 10.5114/biolsport.2022.104917 PMC8919884 35309542

[B38] Mannion A. F. Elfering A. Staerkle R. Junge A. Grob D. Semmer N. K. (2005). Outcome Assessment in Low Back Pain: How Low Can You Go? Eur. Spine J. 14, 1014–1026. 10.1007/s00586-005-0911-9 15937673

[B39] Marković G. Misigoj-Duraković M. Trninić S. (2005). Fitness Profile of Elite Croatian Female Taekwondo Athletes. Coll. Antropol. 29, 93–99. 16117305

[B40] McFarland I. Dawes J. J. Elder C. Lockie R. (2016). Relationship of Two Vertical Jumping Tests to Sprint and Change of Direction Speed Among Male and Female Collegiate Soccer Players. Sports 4, 11. 10.3390/sports4010011 PMC5968930 29910258

[B41] Mejri M. A. Hammouda O. Yousfi N. Haddad M. Souissi N. (2015). “Sleep, Sleep Loss and Performance in Taekwondo Competition,” in Performance Optimization in Taekwondo: From Laboratory to Field. Editor Haddad M. (Heathrow (UK): OMICS International), 1–10.

[B42] Mejri M. A. Yousfi N. Mhenni T. Tayech A. Hammouda O. Driss T. (2016). Does One Night of Partial Sleep Deprivation Affect the Evening Performance during Intermittent Exercise in Taekwondo Players? J. Exerc. Rehabil. 12, 47–53. 10.12965/jer.150256 26933660 PMC4771153

[B43] Menescardi C. Falco C. Ros C. Morales-Sánchez V. Hernández-Mendo A. (2019). Development of a Taekwondo Combat Model Based on Markov Analysis. Front. Psychol. 10, 2188. 10.3389/fpsyg.2019.02188 31632318 PMC6779838

[B44] Mhenni T. Michalsik L. B. Mejri M. A. Yousfi N. Chaouachi A. Souissi N. (2017). Morning-evening Difference of Team-Handball-Related Short-Term Maximal Physical Performances in Female Team Handball Players. J. Sports Sci. 35, 912–920. 10.1080/02640414.2016.1201212 27352917

[B45] Mhenni T. Souissi A. Tayech A. Yousfi N. Mejri M. A. Chamari K. (2021). The Effect of Ramadan Fasting on the Morning-Evening Difference in Team-Handball-Related Short-Term Maximal Physical Performances in Elite Female Team-Handball Players. Chronobiology Int. 38 (4), 1488–1499. [Epub ahead of print]. 10.1080/07420528.2021.1932994 34112026

[B46] Moenig U. (2017). Dominant Features and Negative Trends in the Current World Taekwondo Federation (WTF) Competition System. Ido Mov. Cult. J. Martial Arts Anthrop. 17, 56–67. 10.14589/ido.17.3.7

[B47] Monks L. Seo M.-W. Kim H.-B. Jung H. C. Song J. K. (2017). High-intensity Interval Training and Athletic Performance in Taekwondo Athletes. J. Sports Med. Phys. Fitness 57, 1252–1260. 10.23736/S0022-4707.17.06853-0 28085127

[B48] Negahban H. Aryan N. Mazaheri M. Norasteh A. A. Sanjari M. A. (2013). Effect of Expertise in Shooting and Taekwondo on Bipedal and Unipedal Postural Control Isolated or Concurrent with a Reaction-Time Task. Gait & Posture 38, 226–230. 10.1016/j.gaitpost.2012.11.016 23245642

[B49] Negrete R. Brophy J. (2000). The Relationship between Isokinetic Open and Closed Chain Lower Extremity Strength and Functional Performance. J. Sport Rehabil. 9, 46–61. 10.1123/jsr.9.1.46

[B50] Nimphius S. Callaghan S. J. Bezodis N. E. Lockie R. G. (2018). Change of Direction and Agility Tests: Challenging Our Current Measures of Performance. Strength Condit. J. 40, 26–38. 10.1519/SSC.0000000000000309

[B51] Ojeda-Aravena A. Herrera-Valenzuela T. Valdés-Badilla P. Cancino-López J. Zapata-Bastias J. García-García J. M. (2021). Effects of 4 Weeks of a Technique-specific Protocol with High-Intensity Intervals on General and Specific Physical Fitness in Taekwondo Athletes: An Inter-individual Analysis. Ijerph 18, 3643. 10.3390/ijerph18073643 33807435 PMC8037394

[B52] Ojeda-Aravena A. P. Azócar-Gallardo J. Hérnandez-Mosqueira C. Herrera-Valenzuela T. (2020). Relación entre la prueba de agilidad específica en taekwondo (tsat), la fuerza explosiva y la velocidad líneal en 5-m atletas de taekwondo de ambos sexos (Relationship between the specific agility test in taekwondo (tsat), explosive strength and 5-m linea. Retos 39, 84–89. 10.47197/retos.v0i39.78395

[B53] Park S.-U. Kim D.-K. Ahn H. (2021). A Predictive Model on the Intention to Accept Taekwondo Electronic Protection Devices. Appl. Sci. 11, 1845. 10.3390/app11041845

[B54] Pauole K. Madole K. Garhammer J. Lacourse M. Rozenek R. (2000). Reliability and Validity of the T-Test as a Measure of Agility, Leg Power, and Leg Speed in College-Aged Men and Women. J. Strength Cond. Res. 14, 443–450. 10.1519/1533-4287(2000)014<0443:ravott>2.0.co;2

[B55] Peterson M. D. Alvar B. A. Rhea M. R. (2006). The Contribution of Maximal Force Production to Explosive Movement Among Young Collegiate Athletes. J. Strength Cond. Res. 20, 867–873. 10.1519/R-18695.1 17194245

[B56] Ribeiro A. I. Franchini E. Mesquita P. H. Amaral Junior P. A. Albuqueroqe M. R. (2020). Development and Reliability of a Kick Test System for Taekwondo Athletes. Ido Mov. Cult. J. Martial Arts Anthrop. 20, 31–39. 10.14589/ido.20.4.5

[B57] Salaj S. Markovic G. (2011). Specificity of Jumping, Sprinting, and Quick Change-Of-Direction Motor Abilities. J. Strength Cond. Res. 25, 1249–1255. 10.1519/JSC.0b013e3181da77df 21240031

[B58] Santos J. F. d. S. Dias Wilson V. Herrera-Valenzuela T. Sander Mansur Machado F. (2020). Time-Motion Analysis and Physiological Responses to Taekwondo Combat in Juvenile and Adult Athletes: A Systematic Review. Strength Condit. J. 42, 103–121. 10.1519/SSC.0000000000000517

[B59] Sassi R. H. Dardouri W. Yahmed M. H. Gmada N. Mahfoudhi M. E. Gharbi Z. (2009). Relative and Absolute Reliability of a Modified Agility T-Test and its Relationship with Vertical Jump and Straight Sprint. J. Strength Cond. Res. 23, 1644–1651. 10.1519/JSC.0b013e3181b425d2 19675502

[B60] Sayers M. G. L. (2015). Influence of Test Distance on Change of Direction Speed Test Results. J. Strength Cond. Res. 29, 2412–2416. 10.1519/JSC.0000000000001045 26049789

[B61] Sevinç D. Çolak M. (2019). The Effect of Electronic Body Protector and Gamification on the Performance of Taekwondo Athletes. Int. J. Perform. Anal. Sport 19, 110–120. 10.1080/24748668.2019.1570457

[B62] Sevi̇nç D. (2017). Comparison of the Effects of High Level Technical Strikes of Taekwondo Athletes on the Electronic Head Gear before and after Training. ojrs Vol. 6, 13–27. 10.22282/ojrs.2017.18

[B63] Sheppard J. M. Young W. B. (2006). Agility Literature Review: Classifications, Training and Testing. J. Sports Sci. 24, 919–932. 10.1080/02640410500457109 16882626

[B64] Singh A. Sathe A. Sandhu J. (2017). Effect of a 6-week Agility Training Program on Performance Indices of Indian Taekwondo Players. Saudi J. Sports Med. 17, 139–143. 10.4103/sjsm.sjsm_19_17

[B65] Smith T. B. Hopkins W. G. (2011). Variability and Predictability of Finals Times of Elite Rowers. Med. Sci. Sports Exerc. 43, 2155–2160. 10.1249/MSS.0b013e31821d3f8e 21502896

[B66] Souissi S. Chaouachi A. Burnett A. Hue O. Bouhlel E. Chtara M. (2020). Leg Asymmetry and Muscle Function Recovery after Anterior Cruciate Ligament Reconstruction in Elite Athletes: a Pilot Study on Slower Recovery of the Dominant Leg. bs 37, 175–184. 10.5114/biolsport.2020.94238 PMC7249793 32508385

[B67] Taskin M. Akkoyunlu Y. (2020). Effect of Anaerobic Power on Agility and Quickness in Male National Taekwondo Athletes. Kinesiol. Slov. 26, 49–57.

[B68] Tayech A. Arbi Mejri M. Makhlouf I. Uthof A. Hambli M. G. Behm D. (2022). Reliability, Criterion-Concurrent Validity, and Construct-Discriminant Validity of a Head-Marking Version of the Taekwondo Anaerobic Intermittent Kick Test. bs 39, 951–963. 10.5114/biolsport.2022.109459 PMC9536368 36247969

[B69] Tayech A. Mejri M. A. Chaabene H. Chaouachi M. Behm D. G. Chaouachi A. (2019). Test-retest Reliability and Criterion Validity of a New Taekwondo Anaerobic Intermittent Kick Test. J. Sports Med. Phys. Fitness 59, 230–237. 10.23736/S0022-4707.18.08105-7 29308848

[B70] Tayech A. Mejri M. A. Chaouachi M. Chaabene H. Hambli M. Brughelli M. (2020). Taekwondo Anaerobic Intermittent Kick Test: Discriminant Validity and an Update with the Gold-Standard Wingate Test. J. Hum. Kinet. 71, 229–242. 10.2478/hukin-2019-0081 32148587 PMC7052711

[B71] World Medical Association (2013). World Medical Association Declaration of Helsinki: Ethical Principles for Medical Research Involving Human Subjects. JAMA 310, 2191–2194. 10.1001/jama.2013.281053 24141714

[B72] World Taekwondo (2020). Competition Rules and Interpretation. Available at: http://www.worldtaekwondo.org/rules-wt/rules.html.

[B73] Young W. B. James R. Montgomery I. (2002). Is Muscle Power Related to Running Speed with Changes of Direction? J. Sports Med. Phys. Fitness 42, 282–288. 12094116

[B74] Young W. B. Dawson B. Henry G. J. (2015). Agility and Change-Of-Direction Speed Are Independent Skills: Implications for Training for Agility in Invasion Sports. Int. J. Sports Sci. Coaching 10, 159–169. 10.1260/1747-9541.10.1.159

[B75] Zemková E. Hamar D. (2014). Agility Performance in Athletes of Different Sport Specializations. Acta Gymnica 44, 133–140. 10.5507/ag.2014.013

[B76] Zemková E. Hamar D. (2013). Assessment of Agility Performance under Sport-specific Conditions. AJESS 10, 47–60.

[B77] Zemková E. Hamar D. (2017). Association of Speed of Decision Making and Change of Direction Speed with the Agility Performance. JFNRE 7, 10–15.

