# Supplementary material for: Reliability and Validity of a New Taekwondo-Specific Change-of-Direction Speed Test With Striking Techniques in Elite Taekwondo Athletes: A Pilot Study
Source: Front Physiol. 2022 Apr 26;13:774546. doi: 10.3389/fphys.2022.774546 (PMC9086405; doi:10.3389/fphys.2022.774546)
Supplement: Supplementary file 1 [file Table1.DOCX]

**Supplementary Table│** Sex-specific data of taekwondo athletes (means ± standard deviations) computed with log transformed reliability data of generic performance tests for measuring COD speed, balance, speed, and jump performance.

|  | | Test (s)  (mean ± SD) | Percent change in mean  (90% CL) | Effect size Magnitude  (90% CI) | ICC  Magnitude (90% CL) | TEM  (90% CL)  (%) | SIC_0.2_  (90% CL) (%) |
| --- | --- | --- | --- | --- | --- | --- | --- |
| MTT | Male | 6.70 ± 0.37 | -0.5  (-3.0 to 2.1) | -0.09  Trivial  (-0.82 to 0.65) | 0.71  High  (0.30 to 0.90) | 3.2  (2.3 to 5.3) | 0.9  (-0.3 to 1.3) |
|  | Female | 7.64 ± 0.44 | -3.0  (-4.9 to -1.0) | -0.43  Small  (-1.17 to 0.32) | 0.91  Very high  (0.73 to 0.97) | 2.5  (1.8 to 4.1) | 1.3  (0.5 to 1.8) |
| 5-m sprint | Male | 1.21 ± 0.06 | 1.8  (-0.6 to 4.3) | 0.28  Small  (-0.46 to 1.02) | 0.80  High  (0.48 to 0.93) | 2.9  (2.1 to 4.9) | 1.0  (0.1 to 1.4) |
|  | Female | 1.36 ± 0.12 | 1.2  (-0.2 to 2.5) | 0.17  Trivial  (-0.57 to 0.90) | 0.98  Extremely high  (0.92 to 0.99) | 1.6  (1.2 to 2.7) | 1.7  (0.8 to 2.3) |
| 10-m sprint | Male | 1.99 ± 0.08 | 0.9  (-0.2 to 2.1) | 0.13  Trivial  (-0.61 to 0.86) | 0.91  Very high  (0.74 to 0.97) | 1.4  (1.0 to 2.3) | 0.8  (0.3 to 1.1) |
|  | Female | 2.24 ± 0.16 | 0.6  (-0.5 to 1.6) | 0.06  Trivial  (-0.67 to 0.80) | 0.98  Extremely high  (0.93 to 0.99) | 1.3  (0.9 to 2.1) | 1.5  (0.7 to 2.0) |
| 20-m sprint | Male | 3.36 ± 0.11 | 0.8  (-0.3 to 1.9) | 0.27  Small  (-0.47 to 1.01) | 0.86  Very high  (0.62 to 0.95) | 1.3  (1.0 to 2.2) | 0.6  (0.2 to 0.8) |
|  | Female | 3.72 ± 0.25 | 0.8  (-0.7 to 2.4) | 0.12  Trivial  (-0.62 to 0.85) | 0.95  Extremely high  (0.84 to 0.98) | 1.9  (1.4 to 3.2) | 1.4  (0.5 to 1.8) |
| 30-m sprint | Male | 4.68 ± 0.16 | 0.3  (-0.6 to 1.3) | 0.13  Trivial  (-0.61 to 0.86) | 0.92  Very high  (0.76 to 0.97) | 1.1  (0.8 to 1.8) | 0.6  (0.2 to 0.9) |
|  | Female | 5.25 ± 0.29 | 0.5  (-0.9 to 1.9) | 0.07  Trivial (-0.67 to 0.81) | 0.92  Very high  (0.78 to 0.98) | 1.7  (1.2 to 2.8) | 1.0  (0.4 to 1.4) |
| SJ | Male | 28.1 ± 2.5 | -2.3  (-6.1 to 1.7) | -0.23  Small  (-0.70 to 0.51) | 0.84  Very high  (0.54 to 0.95) | 4.6  (3.3 to 8.1) | 1.8  (-0.2 to 2.5) |
|  | Female | 19.7 ± 2.9 | 1.2  (-3.3 to 5.9) | 0.07  Trivial  (-0.67 to 0.81) | 0.88  Very high  (0.67 to 0.96) | 5.7  (4.1 to 9.5) | 2.7  (0.8 to 3.7) |
| CMJ | Male | 29.6 ± 3.3 | 1.4  (-2.3 to 5.3) | 0.14  Trivial  (-0.59 to 0.88) | 0.91  Very high  (0.73 to 0.97) | 4.3  (3.1 to 7.5) | 2.3  (0.6 to 3.3) |
|  | Female | 22.7 ± 4.7 | -0.8  (-7.6 to 6.5) | 0.10  Trivial  (-0.83 to 0.64) | 0.81  High  (0.51 to 0.94) | 9.1  (6.5 to 15.3) | 3.2  (0.4 to 4.6) |
| SHT | Male | 207.3 ± 28.5 | 1.4  (-0.3 to 2.5) | 0.07  Trivial (-0.67 to 0.80) | 0.99  Extremely high  (0.96 to 1.00) | 1.8  (1.3 to 2.9) | 2.6  (1.2 to 3.5) |
|  | Female | 185.2 ± 16.7 | 0.9  (-1.4 to 3.1) | 0.10  Trivial  (-0.64 to 0.83) | 0.93  Very high  (0.80 to 0.98) | 2.7  (2.0 to 4.6) | 1.8  (0.7 to 2.4) |
| THT | Male | 638.2 ± 82.7 | -1.2  (-5.1 to 3.0) | 0.09  Trivial (-0.83 to 0.64) | 0.89  Very high  (0.68 to 0.96) | 5.1  (3.7 to 8.6) | 2.5  (0.8 to 3.4) |
|  | Female | 519.3 ± 28.8 | -1.5  (-3.1 to 0.1) | -0.28  Small  (-1.02 to 0.46) | 0.89  Very high  (0.69 to 0.96) | 2.0  (1.5 to 3.3) | 1.0  (0.3 to 1.4) |
| 5JT | Male | 11.15 ± 1.43 | 0.9  (-2.1 to 4.1) | 0.05  Small  (-0.69 to 0.78) | 0.92  Very high  (0.78 to 0.98) | 3.8  (2.7 to 6.3) | 2.3  (0.8 to 3.1) |
|  | Female | 9.05 ± 0.49 | 2.1  (-0.5 to 4.7) | 0.35  Small  (-0.39 to 1.10) | 0.78  High  (0.44 to 0.93) | 3.2  (2.3 to 5.2) | 1.0  (-0.1 to 1.5) |
| SST | Male | 4.66 ± 2.16 | -0.2  (-10.3 to 11.0) | -0.04  Small  (-0.78 to 0.69) | 0.95  Extremely high  (0.85 to 0.98) | 13.9  (10.0 to 23.9) | 10.1  (4.1 to 13.9) |
|  | Female | 3.69 ± 0.77 | -1.0  (-6.0 to 4.2) | -0.04  Small  (-0.77 to 0.70) | 0.94  Very high  (0.82 to 0.98) | 6.5  (4.7 to 10.9) | 4.3  (1.7 to 5.9) |
| YBT-CS | Male | 101.01 ± 7.39 | 0.02  (-1.7 to 1.7) | 0.00  Small  (-0.74 to 0.74) | 0.94  Very high  (0.83 to 0.98) | 2.1  (1.5 to 3.4) | 1.5  (0.6 to 2.0) |
|  | Female | 98.00 ± 10.05 | 1.0  (-0.7 to 2.8) | 0.10  Small  (-0.64 to 0.84) | 0.97  Extremely high  (0.91 to 0.99) | 2.1  (1.5 to 3.5) | 2.0  (0.9 to 2.7) |

*MTT: modified* COD *T-test; SJ: squat jump test; CMJ: countermovement jump test; SHT: single‐leg hop test; THT: single-leg triple hop test; 5JT: 5-jump test; SST: standing stork balance test; YBT-CS: Y-balance-composite score;SD: standard deviation; 90% CI: 90% compatibility interval; 90% CL: 90% compatibility limits; ICC: intraclass correlation coefficient; TEM: Typical error of measurement as coefficient of variation (CV) (%);SIC_0.2_: Smallest important change based on 0.2 of the between-athlete SD; times/divide factor: ×/÷1.1.*
